# Candidiasis epidemiology and outcomes including emergence of *Candida auris* from a large, Southern US metro area: a six-year evaluation

**DOI:** 10.1017/ash.2025.10200

**Published:** 2025-11-03

**Authors:** Ricky Huynh-Phan, Ardath Plauche, William L. Musick, Kady Phe, Wesley J. Hoffman, Mayar Al Mohajer, Todd Lasco, Nicholas D. Beyda, Taryn A. Eubank, Kevin W. Garey

**Affiliations:** 1 University of Houston College of Pharmacy, Houston, TX, USA; 2 Baylor St. Luke’s Medical Center, Houston, TX, USA; 3 Memorial Hermann Health System, Houston, TX, USA; 4 Houston Methodist Hospital, Houston, TX, USA; 5 Baylor College of Medicine, Houston, TX, USA

## Abstract

**Background:**

Invasive candidiasis including candidemia is a common healthcare-associated infections with significant morbidity and mortality. The USA does not have mandatory national surveillance for mucocutaneous or invasive candidiasis which complicates estimation of epidemiology and outcomes. The aim of this project was to describe the epidemiology, mortality, and *Candida*-associated hospital readmissions in hospitalized patients with *Candida* species infections.

**Methods:**

This secondary database analysis used clinical microbiology data from adults hospitalized at three large health systems (25-hospitals) in the Greater Houston area totaling over 1.6 million hospitalization days per year from 2018 to 2023. Proportion and rates of Candida cultures per 10,000 hospitalization days were calculated. Risk factors for mortality and *Candida*-associated readmissions were assessed by multivariable logistic regression.

**Results:**

Within the study period, 7514 hospitalized patients aged 64 ± 16 years (mean± standard deviation (SD)) with 10,183 unique *Candida* cultures were identified. Majority of *Candida* cultures were nosocomial (59%) with wide variability in mean time to positive culture (9 ± 44 days) after admission. *Candida* specimens were from blood (32%), abdomen (29%), or mucocutaneous (24%) cultures and most commonly *C. albicans* (44%) or *C. glabrata* (21%). *C. auris* increased significantly from 2% of cultures from 2018–20 to 5% in 2021–23 (*p* < 0.0001). Length of hospital stay was 21 ± 34 days and inpatient mortality was 17%. Multivariable analyses identified hospitalization variables and *Candida* species predictive of inpatient all-cause mortality and *Candida*-associated readmissions after initial hospitalization.

**Conclusion:**

These analyses highlight the significant burden of candidiasis and the emergence of new strains, including *C. auris*. Ongoing surveillance can refine burden estimates and assess the impact of stewardship and infection control interventions.

## Introduction

Invasive candidiasis is one of the most common healthcare-associated infection in the United States associated with significant all-cause inpatient mortality.^1^ The burden of disease is high in hospitalized patients with approximately 100 cases per 100,000 admissions with *Candida* species accounting for around three percent of all bloodstream infections.^2,3^ Pathomechanism of disease involves overgrowth of yeast in the mouth, intestine, and other mucosal surfaces generally due to antibiotic use allowing selection for oropharyngeal, vulvovaginal, or cutaneous candidiasis.^4^ Disruption of the intestinal barrier or breach in skin integrity causes dissemination of invasive candidiasis disease to the blood, abdomen, brain, and elsewhere. *Candida albicans* is the most isolated *Candida* species, although non-albicans species including *Candida auris* continue to increase. Unlike other healthcare-associated infections, candidiasis is associated with a late recurrence of infection defined as at least 30 days after the last positive *Candida* species culture for a prior case.^5^ Late recurrence occurs in 2%–17% of patients with most studies focused on candidemia.^6,7^

The USA does not have mandatory national surveillance for candidiasis which complicates estimation of mucocutaneous or invasive candidiasis epidemiology and outcomes. The 2016 IDSA Candidiasis Guidelines emphasizes the understanding local epidemiology being critical in making informed therapeutic decisions.^8^ To meet this unmet need, the Centers for Disease Control and Prevention (CDC) Emerging Infections Program (EIP) recently published population-based active surveillance for culture-confirmed candidemia from 10 sites across the USA from 2017 to 2021.^9^ However, Texas was not part of the 10 EIP sites providing a unique opportunity to compare EIP results to the Greater Houston area, an urban area of approximately 5 million persons that includes the metro city of Houston, Texas. Using data from clinical microbiology departments of three large health systems, the aim of this project was to describe the epidemiology, mortality, and *Candida*-associated readmissions in hospitalized patients with mucocutaneous or invasive candidiasis.

## Methods

### Data source and study population

This secondary database analysis utilized data from three large health systems in the Greater Houston area, comprising three tertiary care hospitals and 22 community hospitals totaling over 1.6 million hospitalization days annually. Data from 2018 to 2023 for hospitalized patients over the age of 18 was used for these analyses. The study was conducted following the Declaration of Helsinki and was approved by the University of Houston Committee for the Protection of Human Subjects (CPHS: 00002244) and associated hospitals.

### Data collection and definitions

Using the electronic health records of each health system, a unique patient study identifier, age, admit date, discharge date, discharge disposition (dead/alive), hospital type (tertiary care/community), culture date, culture source, and *Candida* species was obtained from the clinical microbiology laboratory for all positive *Candida* cultures during the study period. This was a non-interventional study, all cultures were obtained at the discretion of the hospital treating team and samples were processed as part of the hospital’s clinical microbiology laboratory standard of care. Surveillance cultures were excluded. Data were cleaned by standardizing *Candida* species names and culture sources. Anamorph names were utilized throughout this study in accordance with recommendations for laboratory reporting and ease of comparison to other surveillance studies (Supplementary table 1).^10^ Culture sources were categorized as mucocutaneous infection (including oropharyngeal, vulvovaginal, and cutaneous candidiasis) or invasive candidiasis (including candidemia, abdominal candidiasis, other organ systems such as brain, lung, heart, and eyes). Due to the feasibility of distinguishing bone cultures from cutaneous cultures, bone cultures were included in mucocutaneous candidiasis. Duplicate *Candida* species from the same culture source obtained on the same culture day were removed. Nosocomial cultures were defined as any positive *Candida* cultures obtained more than 3 days after hospital admission. Inpatient mortality was defined as all-cause mortality that occurred during the index hospitalization. *Candida*-associated readmission was defined as a subsequent hospitalization with a positive culture results for a *Candida* species from any source anytime during the subsequent re-hospitalization. Follow-up for readmission was limited to the study period.

### Analysis

Descriptive statistics including mean ± SD for the index admission with a positive *Candida* culture were used to describe the number of patients and samples, hospital type, patient age, days from admission to date of first *Candida* culture, time from admission to all *Candida* cultures, sample source, *Candida* species, length of hospital stay, inpatient mortality, and *Candida*-associated readmission. The proportional change in cultures from community and tertiary hospitals, as well as culture source over time was compared using the Mantel-Haenszel χ^2^ test. Rates per 10,000 hospitalization days were calculated for discharge mortality, *Candida* species and source of cultures were compared by source (mucocutaneous vs invasive) and hospital type (tertiary vs community) using a Student’s *t*-test, respectively. Univariate statistics (Chi square and Student t-test) were performed for inpatient mortality and *Candida*-associated readmissions. Two separate multivariable logistic models were built to identify risk for discharge mortality (model 1) and *Candida*-associated readmission (model 2) including hospital type (tertiary/community), *Candida* species, source, year of collection, patient age, and any other variable with a *P* < .2 from the univariate analysis were included as independent variables in the model. For patients with multiple positive cultures, only the first *Candida* culture was used in these models. Statistical analysis was performed using SAS Version 9.3 (SAS Institute, Cary, NC). A *P* < .05 was considered significant. Data visualization was performed using R and R Studio (PBC, Boston, MA) or NCSS 2022 (NCSS, LLC, Kaysville, Utah).

## Results

### Demographics and epidemiology

**Figure 1.**
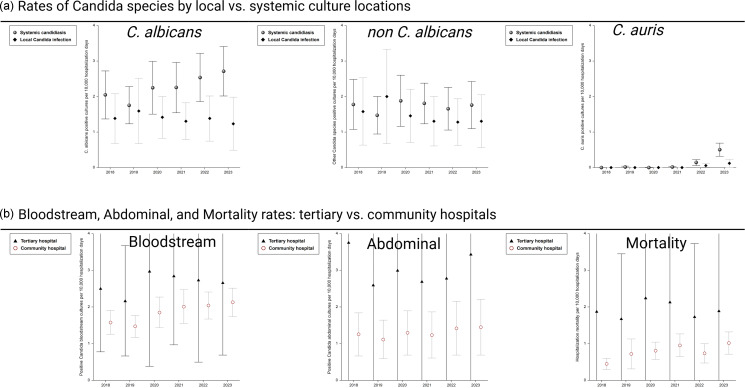
*Candida* culture rate data for patients with first admission. Rates represent initial hospitalization and first culture of each *Candida* species isolated during the initial hospitalization. Local Candidiasis: oropharyngeal, vulvovaginal, and cutaneous Candidiasis; invasive candidiasis: Candidemia, abdominal Candidiasis, other organ systems (brain, lung, heart, and eyes).

Seven thousand five hundred and fourteen patients aged 64 ± 16 years with 10,183 unique *Candida* cultures were identified during the study period. The majority (59%) of cultures were nosocomial, with a mean positive culture grown at 9 ± 44 days after hospital admission. Overall length of hospital stay was 21 ± 34 days. Majority of positive *Candida* specimens were from the blood (32%) followed by abdomen (29%), cutaneous (24%), oropharyngeal (6%), or other sources (6%). The most common *Candida* species were *C. albicans* (44%), *C. glabrata* (21%), *C. parapsilosis* (13%), *C. tropicalis* (9%), and *C. auris* (3%). Changes in demographics and fungal cultures between 2018–2020 and 2021–23 are shown in Table 1. Proportion of cultures from community hospitals increased during the later time as did the proportion of cultures positive from blood samples versus other sources (*P* < .0001, each). *C. auris* increased significantly from 2% of cultures from 2018–20 to 5% in 2021–23 (*P* < .0001). Rates of *C. albicans* and non-*C. albicans* positive cultures were higher from invasive candidiasis cultures (2–3 positive cultures per 10,000 patient days) compared to mucocutaneous candidiasis cultures (1–2 positive cultures per 10,000 patient days; *P* < .001). *C. auris* was detected starting in 2021 and by 2023 averaged 0.12 (95% CI: 0.00–0.23) positive cultures per 10,000 patient days from local cultures and 0.51 (95% CI: 0.32–0.69) positive cultures per 10,000 patient days from systemic cultures. Rates of bloodstream and abdominal *Candida* cultures per 10,000 patient days were higher in tertiary hospitals (2–4 positive cultures per 10,000 patient days) compared to community hospitals (1–2 positive cultures per 10,000 patient days; *P* < .001). Inpatient mortality was also significantly higher in tertiary hospitals (1.93 [95% CI: 1.37–2.49] deaths per 10,000 patient days) compared to community hospitals (0.78 [95% CI: 0.67–0.89] deaths per 10,000 patient days; *P* < .001). *Candida*-associated rates are shown in Figure 1.

**Table 1.** Patient demographics, hospitalization data, and *candida* species data over time

	Total		2018–2020		2021–23		
	N	%	N	%	N	%	*p* value
**Number of unique patients**	7 514	N/A	3 557	N/A	3 957	N/A	N/A
**Number of samples**	10 183	N/A	4 827	N/A	5 356	N/A	N/A
**Unique patient by hospital type**							
**Community hospitals**	3 350	55%	1503	42%	1847	47%	.0001
**Tertiary care hospitals**	4 164	45%	2 054	58%	2 110	53%	
**Age (mean +/-SD)**	64 ± 16		65 ± 16		63 ± 16		<.0001
**Days from admission to first *Candida* culture**	9 ± 44		9 ± 61		8 ± 17		.69
**Nosocomial cultures**	6 026	59%	2 795	58%	3 231	60%	.01
**Sample source**							<.0001
**Blood**	3 267	32%	1 408	29%	1859	35%	
**Abdomen**	2 930	29%	1 414	29%	1516	28%	
**Lung**	314	3%	145	3%	169	3%	
**Oropharyngeal**	570	6%	316	7%	254	5%	
**Cutaneous**	2 448	24%	1 234	26%	1 214	23%	
**Other**	654	6%	310	6%	344	6%	
***Candida* species**							<.0001
***C. albicans***	4 486	44%	2 224	46%	2 262	42%	
***C. glabrata***	2 134	21%	986	20%	1 142	21%	
***C. parapsilosis***	1 373	13%	671	14%	702	13%	
***C. tropicalis***	891	9%	433	9%	458	9%	
***C. auris***	266	3%	7	2%	259	5%	
**Length of hospital stay, days**	21 ± 34		20 ± 38		21 ± 29		.26
**Discharge mortality**	1 251	17%	556	16%	695	18%	.02
***Candida*-associated readmissions**	597	10%	328	11%	296	9%	.0001

**Figure 2.**
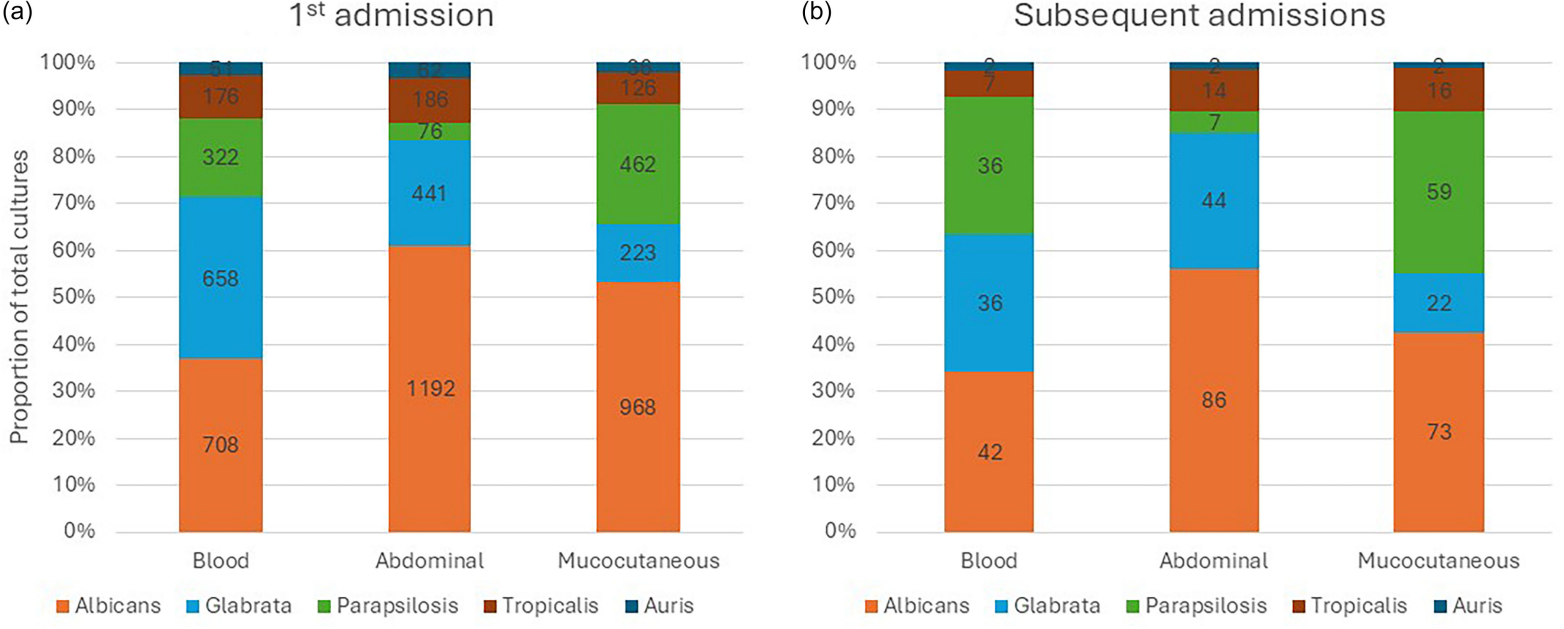
*Candida* species and source in hospitalized patients with first (panel A) or subsequent admissions (panel B).

### Mortality rates

One thousand two hundred and fifty-one patients of 7514 (17%) patients with positive *Candida* cultures died during their hospitalization. Inpatient mortality was lower at community (0.5–1 deaths per 10,000 patient days) compared to tertiary (1.5–2.5 deaths per 10,000 patient days) hospitals. Inpatient mortality was significantly higher in patients with nosocomial (23%) compared to non-nosocomial (10%) cultures (*P* < .0001), invasive candidiasis (23%) compared to mucocutaneous candidiasis (7%) (*P* < .0001), bloodstream (36%) versus non-bloodstream (9%) culture sources (*P* < .0001), and *C. glabrata* (20%), *C. tropicalis* (21%), *C. krusei* (27%), or *C. auris* (30%) compared to other *Candida* species (*p* ≤ .005, each). In multivariable analysis (Table 2), admission to a tertiary care hospital (OR: 1.2; 95% CI: 1.06–1.39; *P* = .0046), nosocomial cultures (OR: 2.6; 95% CI: 2.3–3.0; *P* < .0001), blood culture (OR: 5.8; 95% CI: 5.1–6.7; *P* < .0001), *C. tropicalis* (OR: 1.3 95% CI: 1.0–1.6; *P* = .02), and *C. krusei* (OR:1.8; 95% CI: 1.1–3.0; *P* = .02) were significant independent predictors of inpatient mortality.

**Table 2.** Predictors of inpatient mortality and *candida*-associated readmissions in 7 514 hospitalized patients; results from multivariable regression analysis. Blanks represent variables not found to be significant in multivariable analysis. All other candida species used as reference for the *candida* species evaluated below

	Inpatient mortality			*Candida*-associated readmission		
Variable	Point Estimate	95% Wald Confidence Limits	*p* value	Point estimate	95% Wald Confidence Limits	p value
Age, years				.98	.97–.99	<.0001
Admitted to a tertiary care hospital	1.21	1.06–1.39	.00	1.56	1.30–1.88	<.0001
Nosocomial culture	2.61	2.28–3.00	<.0001			
Blood culture	5.84	5.11–6.68	<.0001			
*C. glabrata*				1.29	1.03–1.61	.03
*C. tropicalis*	1.31	1.04–1.65	.02			
*C. parapsilosis*				1.66	1.30–2.12	<.0001
*C. krusei*	1.81	1.09–3.01	.02	2.17	1.10–4.32	.03
*C. auris*				2.12	1.21–3.72	.01

### Candida-associated readmission

Five hundred and ninety-seven of 6,263 discharged patients (9.5%) were readmitted with positive *Candida* cultures during the study period. Patients were readmitted a median of 98 (IQR: 26–351) days after discharge from the index hospitalization. One hundred fifty-three of 597 patients (26%) were hospitalized within 30 days of discharge from their index hospitalization. Of the 597 patients readmitted with positive *Candida* cultures, 485 (89%) were discharged alive after the readmission. Excluding those who died during their index hospitalization, patients who were readmitted with positive *Candida* cultures were younger (61 ± 15 yr) than those not readmitted (64 ± 16 yr; *P* = .0001), more likely to be initially hospitalized at a tertiary hospital (10%) versus a community hospital (7%; *P* < .0001), and more likely to have non-*C. albicans* cultures (51%) compared to *C. albicans* (45%) cultures during their index hospitalization (*P* = .017). In multivariable analyses (Table 2), younger age (OR: 0.99; 95% CI: 0.98-0.99; *P* < .0001), admission to a tertiary care hospital (OR: 1.56; 95% CI: 1.30–1.88; *P* < .0001), and non-*C. albicans* species including *C. glabrata* (OR: 1.29; 95% CI: 1.03–1.61; *P* = .030), *C. parapsilosis* (OR: 1.66; 95% CI: 1.30–2.12; *P* < .0001), *C. krusei* (OR: 2.17; 95% CI: 1.1–4.3; *P* = .027), and *C. auris* (OR: 2.12; 95% CI: 1.21–3.72; *P* = .0088) were significant independent predictors of *Candida*-associated readmissions after index hospitalization.

## Discussion

Surveillance studies are of the utmost importance for detecting emerging pathogens and serve as a cornerstone of public health. The only national surveillance study in the USA is the CDC EIP. This group recently published population-based estimates for culture-confirmed candidemia from 2017–2021 demonstrating an overall incidence of 7.4 cases per 100,000 population with considerable variation noted between population areas.^9^ Identifying 6,235 patients and a total of 7381 candidemia cases, *C. albicans* was most isolated (37%) followed by *C. glabrata* (30%), *C. parapsilosis* (14%), and *C. tropicalis* (6%). *C. auris* was identified in 0.4% of cases. Median length of hospitalization was 16 days (IQR: 7–33 d), and 33% of cases involved in-hospital deaths with a range of 27%–36%. These data represent the best population-based estimates of invasive candidiasis but are limited to the specific sites used by the CDC EIP network.

In our current study, we integrated clinical microbiology data from 25 tertiary and community hospitals in the Greater Houston area to identify a similar distribution of *Candida* species among hospitalized patients with candidiasis including bloodstream infections and mucocutaneous infections. Inpatient all-cause deaths in cases with *Candida* cultures in the bloodwere 36%, comparable to the CDC EIP candidemia study findings. Findings from our study were obtained during a similar time frame with results comparable to the CDC EIP data, again noting increased incidence and mortality in the COVID-19 pandemic era. These analysis techniques could provide a method to generate comparison data in areas of the USA not covered by the EIP program.

Late recurrent candidiasis has been described in case reports and series and is generally defined as a second case of candidiasis occurring at least 1-month after the last blood culture positive with *Candida* species was obtained from the initial case.^11^ Using a statewide database, the Connecticut Department of Health in conjunction with the CDC EIP program evaluated readmissions due to candidemia in 347 cases of index candidemia.^12^ Excluding 121 (35%) patients who died during the index admission, 128 of the remaining 226 individuals (57%) were readmitted for any reason during the follow-up period of which the majority (75%) were admitted within 30-days. Hospital-onset candidemia was the sole independent predictor of all-cause readmission. Our study significantly contributes to these data by demonstrating that most *Candida*-associated readmissions occur after 30-days of prior discharge. Type of species (primarily non-*C. albicans*) and admission to a tertiary hospital were more important predictors than hospital-onset but this may be due to more granular data in our database as well as evaluation of candidemia and other *Candida* species infections. Although all these findings are limited by geographic areas, it is interesting to note the consistency of these findings amongst different geographic areas.

*C. auris* has quickly become a prevalent multidrug-resistant pathogen with high infection control concerns. *C. auris* incidence increased over time with rates of 0.5 cases per 10,000 patient days from systemic culture sources. This data provides important hospitalization rates to benchmark continued emergence of *C. auris* at ours or other geographic locations. An integrated health system in Miami, Florida also showed increased volume of clonal *C. auris* blood cultures mostly belonging to the South African clade III strains.^13^ Continued genotypic and phenotypic surveillance of this emerging multidrug-resistant pathogen will be crucial to infection control and patient management in the future.

These findings have notable limitations. Each health system microbiology database produced different reports and required significant cleaning to be incorporated together. Species confirmation was not confirmed beyond normal clinical microbiology processes; some misclassification in Candida species might have occurred. Trained investigators used consistent definitions but inconsistencies in data interpretation are still possible. We extracted data from the clinical microbiology departments only, and therefore, other important data such as antifungal treatment were not collected. Expansion of our data collection will be a future focus. However, using the data we obtained, we can replicate many of the analyses from CDC EIP data with a contemporary database. Obtaining these isolates for future susceptibility and genomic analyses will also be prioritized. Finally, blood and abdominal cultures are predictive of invasive candidiasis but have low sensitivity in certain cases potentially making our rates being lower than the actual incidence.

In conclusion, these analyses underscore the substantial burden of candidiasis and the emergence of novel strains, including *C. auris*. Ongoing surveillance can refine burden estimates and assess the impact of stewardship and infection control interventions.

## Supporting information

10.1017/ash.2025.10200.sm001 Huynh-Phan et al. supplementary material Huynh-Phan et al. supplementary material

## Supplementary material

The supplementary material for this article can be found at https://doi.org/10.1017/ash.2025.10200

## References

[1] Magill SS , Edwards JR , Bamberg W , et al. Multistate point-prevalence survey of health care-associated infections. N Engl J Med 2014;370:1198– 1208. 24670166 10.1056/NEJMoa1306801 PMC4648343

[2] Bourassa-Blanchette S , Biesheuvel MM , Lam JC , et al. Incidence, susceptibility and outcomes of candidemia in adults living in Calgary, Alberta, Canada (2010–2018). BMC Infect Dis 2023;23:100. 36803357 10.1186/s12879-023-08050-0 PMC9940426

[3] Koehler P , Stecher M , Cornely OA , et al. Morbidity and mortality of candidaemia in Europe: an epidemiologic meta-analysis. Clin Microbiol Infect 2019;25:1200– 1212. 31039444 10.1016/j.cmi.2019.04.024

[4] Lass-Florl C , Kanj SS , Govender NP , Thompson GR , 3rd, Ostrosky-Zeichner L , Govrins MA. Invasive Candidiasis. Nat Rev Dis Primers 2024;10:20. 38514673 10.1038/s41572-024-00503-3

[5] Lai MY , Hsu JF , Chu SM , et al. Risk factors and outcomes of recurrent Candidemia in children: relapse or re-infection? J Clin Med 2019;8:99. 30654524 10.3390/jcm8010099 PMC6352033

[6] Munoz P , Vena A , Valerio M , et al. Risk factors for late recurrent Candidaemia. A retrospective matched case-control study. Clin Microbiol Infect 2016;22:277 e211–220. 10.1016/j.cmi.2015.10.023 26548507

[7] Ala-Houhala M , Anttila VJ. Characteristics of late recurrent Candidemia in adult patients. Mycoses 2021;64:503– 510. 33377571 10.1111/myc.13236

[8] Pappas PG , Kauffman CA , Andes DR , et al. Clinical practice guideline for the management of Candidiasis: 2016 update by the Infectious Diseases Society of America. Clin Infect Dis 2016;62:e1– 50. 26679628 10.1093/cid/civ933 PMC4725385

[9] Jenkins EN , Gold JAW , Benedict K , et al. Population-based active surveillance for culture-confirmed candidemia - 10 sites, United States, 2017–2021. MMWR Surveill Summ 2025;74:1– 15. 10.15585/mmwr.ss7404a1 PMC12115505 40424200

[10] Kidd SE , Abdolrasouli A , Hagen F. Fungal Nomenclature: Managing change is the name of the game. Open Forum Infect Dis 2023;10:ofac559. 36632423 10.1093/ofid/ofac559 PMC9825814

[11] Clancy CJ , Barchiesi F , Falconi DiFrancesco L , et al. Clinical manifestations and molecular epidemiology of late recurrent Candidemia, and implications for management. Eur J Clin Microbiol Infect Dis 2000;19:585– 592. 11014620 10.1007/s100960000335

[12] Suschana E , Correa MA , Meek J , Banach DB. An evaluation of outcomes and hospital readmissions among individuals with Candidemia using statewide surveillance. Infect Control Hosp Epidemiol 2024;45:998– 1002. 38561197 10.1017/ice.2024.52

[13] Rosa R , de Paula Baptista R , Tran TT , et al. Changing trends in the sources and volumes of clinical cultures with Candida auris at an integrated health system in Miami, Florida, United States, 2019–2023. Am J Infect Control 2025;53:719 –722. 40107456 10.1016/j.ajic.2025.03.013 PMC13141654

