# Supplementary material for: Candidiasis epidemiology and outcomes including emergence of Candida auris from a large, Southern US metro area: a six-year evaluation
Source: Antimicrob Steward Healthc Epidemiol. 2025 Nov 3;5(1):e296. doi: 10.1017/ash.2025.10200 (PMC12616563; doi:10.1017/ash.2025.10200)
Supplement: Huynh-Phan et al. supplementary material [file S2732494X25102003sup001.pdf]

Supplemental Table 1.

**Anamorph name**	**Teleomorph name**
Candida albicans	*Candida albicans*
Candida glabrata	***Nakaseomyces glabratus***
Candida tropicalis	*Candida tropicalis*
Candida parapsilosis	*Candida parapsiosis*
Candida krusei	***Pichia kudriavzevii***
Candida auris	*Candida auris*
Candida lusitaniae	***Clavispora lusitaniae***
Candida guilliermondii	***Kodamaea ohmeri***
Candida rugosa	***Diutina rugosa***

Bolded are utilized names by the International Code of Nomenclature for algae, fungi, and plants
